# Hospitals and Their Expenditure

**Published:** 1889-05-18

**Authors:** 


### Hospitals and their Expenditure.

For a study of the tables given in "The Hospital Annual," to which we have already drawn attention,* two things appear—first, that, as has been pointed out, provincial, Scotch, and Irish hospitals are more economically managed than those of the metropolis; and secondly, that, in London and elsewhere, a hospital with a medical school attached costs more than one without. No reason is given for this fact, but it is not difficult to form a surmise. In an institution whose wards are made use of for clinical teaching, there is, and must be, a desire on the part of the honorary physicians and surgeons to collect those cases which present features of exceptional interest. It is true that the average medical practitioner does not in his daily work come across many of the rare and obscure diseases that are found within the hospital walls; but he may meet them any day, and it is necessary that in his student career he should be taught to recognise and treat them. He will have more to do with pneumonia than with parenchymatous nephritis, but he must learn the diagnosis of both, and as three cases of pneumonia will teach him as much as thirty, the wards of the hospital cannot be filled with pneumonia to the disregard of other ailments. We are conscious that in making this statement we are giving an opening to the enemies of hospitals to say, "This, then, is a medical charity! The sufferings of the sick poor are of no account, as compared with the gratification of clinical curiosity."

We must beg those who would thus exclaim to argue the matter reasonably. The situation is parallel to that of the privileges accorded in so many hospitals to subscribers of a certain amount. Anyone who gives a guinea a year will receive as a *quid pro quo* the right to recommend a certain number of patients during that time. The student gives a donation of ten or twelve guineas to a hospital on the understood condition that he receives as a *quid pro quo* a certain amount of material for clinical study. Withdraw the material and the student withholds his donation, a proceeding manifestly to the disadvantage both of the hospital and the sick poor.

But these rare diseases demand treatment which is often expensive, because it is experimental. The word experiment may be objected to; but surely there cannot be many so stupid or prejudiced as to believe that an "experiment" in medicine means the haphazard exhibition of one thing after another, without method or reason. A medical experiment is, indeed, the trial of a new mode of treatment, but that treatment is founded on a careful study of the constitutional and local symptoms of the case, and is guided by the strictest scientific deductions. And as every generation evolves new diseases and new complications of old ones, this patient and conscientious experiment must go on. As we have said, such treatment is expensive. It often involves the importation of new drugs, the special manufacture of new splints, dressings, and apparatus, some of which will ultimately be superseded; for it is rash to guarantee success for any human effort. This point, however, is clear, that the nature of the cases treated in hospitals with medical schools attached to them causes the expenditure on surgery and dispensary to be larger than elsewhere. Of course different hospitals present considerable variety in this matter. Middlesex, for example, spends £8 1s. 2d. annually in this department for each occupied bed, while at University College the amount is £17 5s. 1d. per occupied bed. One cannot help wondering why the difference is so great, though it may be partly accounted for by the fact that University College passes fifteen patients through each bed in the course of the year, while Middlesex shows a proportion of only ten to a bed. Length of stay frequently implies a comparative saving. Still, it cannot of itself explain for the expenses under this head at one institution being twice as great as at the other.

Again, on the surgical side, there are in hospitals with medical schools attached to them an unusually large number of serious operations. The doctors at these hospitals are usually men of considerable repute, to whom severe cases of every kind are brought from town and country. These cases, especially when they require operative treatment, demand constant and skilful nursing. Sometimes they are not left alone a moment for several days, and the nurses must be trained to note every change of temperature, every variation of pulse-beat, for these things indicate to which side victory inclines in the battle between science and death. This means, of course, that the staff of nurses shall be large and thoroughly trained.

In some respects a nurse in a hospital with a medical school may have rather less to do. Surgical dressings which might elsewhere, and in private work, be entrusted to her, are done by the students. But in the scientific work of nursing as distinguished from medicine—we mean the practice of constant and intelligent observation, the management of food and the administration of medicine, the proper use of the numerous appliances for a patient's comfort which have been introduced during recent years—in all these things the work of a nurse in a teaching hospital demands both talent and training. And, as we have said, the staff must be large. The number of nurses that would be excessive in a workhouse infirmary, where the inmates suffer mostly from chronic complaints and the debility of old age, would be insufficient for the work of a general hospital of equal size.

While admitting that the cost of the surgery and dispensary department must be greater in hospitals to which medical schools are attached than in others, we must express our surprise that the expense under this heading is so much greater in London than elsewhere. Drugs and appliances are not dearer in the metropolis than in Edinburgh or Birmingham; yet, with only three exceptions (St. George's, Middlesex, and Westminster), the lowest cost per occupied bed for these things in London exceeds the highest in the hospitals connected with the other medical schools of the kingdom. At the Queen's Hospital, Birmingham, the expenditure under this head is £9 12s. 3d. per occupied bed; and this is the largest sum we meet with. Yet there has been good work done in Birmingham—successes in medical science that cannot be ignored. At the Royal Infirmary, Edinburgh, with its large and important clinical school, the expenditure is £5 19s. 9d. per occupied bed; and this is extravagant compared with Glasgow, where the proportions are £5 8s. 7d. at the Royal Infirmary, and £4 18s. 10d. at the Western Infirmary. The last is the lowest expenditure found anywhere. The three Scotch hospitals quoted are certainly very large, having 700, 542, and 400 available beds respectively; and as we have frequently pointed out, size makes for economy—though perhaps less in this department of hospital expenditure than any other; but the Queen's Hospital has only 120 beds. It is thus smaller than any of the London teaching hospitals, yet it alone approaches their average expenditure in this department.

We have devoted perhaps too much space to this department, because there is no mistaking what the phrase "surgery and dispensary" means. It cannot be stretched to include the porter's uniform, nor narrowed to exclude carbolic gauze. In others items it is not so easy to form a judgment from figures. When we see the charges for provisions, we want to know if this includes the total board of the patients and resident staff, or if the former are expected to provide some things—almost necessaries, such as tea, sugar, etc.—themselves. Under the heading "Alcohol" we should like to know if the sum set down applies to patients only, or if it includes any provision of the sort for house surgeons and nurses. Without being sure that the circumstances are exactly alike, it is unjust to form comparisons. On the other points we can but surmise that the officials do not interpret the terms in the same way. Otherwise we cannot understand how "domestic expenses" amount to only £41 7s. 2d. per occupied bed at St. George's, and to £20 10s. 5d. at the Middlesex. When, however, we go from special items to the total expenditure we can hardly

* See *The Hospital*, Vol. VI., p. 80.

be deceived as to which hospitals are striving for economy. Things omitted under one heading have been included under another, and the total expenditure gives a just view of all. Thus judged, in the London hospitals the two that are nearest in size give the extremes in cost on either side. King's College, with 206 available beds, has an average cost per occupied bed of £167 14s. 7d.; while Westminser, with 205, amounts only to £71 13s. 1d.—less than half. It is true, however, that part of King's College has a smaller average of occupied beds than Westminster—151 to 172; but as the higher expenditure is observable in items where a smaller number of patients would cause a reduction, such as provisions, surgery, and dispensary, it cannot be explained away to any appreciable degree by the smaller number of patients treated.

Judged by this standard also, the provinces show as more economical than London. The highest total expenditure is at the Bristol Royal Infirmary—£81 18s., which is greater than that of Guy's, St. Mary's, and Westminster, but smaller than all the other metropolitan teaching hospitals. No other hospital approaches the London rate; the next highest to Bristol being the Queen's Hospital, Birmingham, where the average per occupied bed is £67 8s. 1d. But Glasgow certainly bears the palm in this matter, the rate at the Royal Infirmary being £47 9s. 3d., and at the Western £49 0s. 3d. Surely, London has something to learn.

